# Prognostic Impact of the Gustave Roussy Immune Score on Cancer-Specific Survival and Treatment Completion in Patients with Bladder Cancer

**DOI:** 10.3390/diagnostics16040574

**Published:** 2026-02-14

**Authors:** Neslihan Özyurt, Aykut Turhan

**Affiliations:** Department of Medical Oncology, Ordu University Training and Research Hospital, Altınordu 52200, Ordu, Turkey; dr.aykutturhan@gmail.com

**Keywords:** bladder cancer, Gustave Roussy Immune Score, GRIm-score, cancer-specific survival, systemic inflammation, treatment completion

## Abstract

**Background**: Bladder cancer has diverse clinical outcomes, even in patients receiving curative treatment. Traditional clinicopathological indicators inadequately assess individual risk. The Gustave Roussy Immune Score (GRIm-score), which combines albumin, lactate dehydrogenase (LDH), and neutrophil-to-lymphocyte ratio (NLR), is a prognostic factor for solid tumors; however, its role in bladder cancer remains unclear. **Methods**: In this retrospective cohort study, patients with bladder cancer confirmed through histopathology received bladder-preserving multimodal therapy or radical cystectomy between October 2010 and April 2025. Participants were grouped into low (0–1) and high (2–3) GRImS categories for analysis. The study examined cancer-specific survival (CSS) and the secondary outcomes of progression-free survival (PFS), overall survival (OS), and treatment completion. Survival analyses were performed using the Kaplan–Meier method and Cox proportional hazard regression. **Results**: A total of 89 patients participated in the study, with 73 (82.0%) and 16 (18.0%) having low and high GRIm-Score. During a median follow-up of 21.5 months, patients with a high GRIm-score had significantly shorter PFS, OS, and CSS than those with a low GRIm-score. The median CSS was 14.07 months for the high GRIm-score group and 27.75 months for the low GRIm-score group (*p* = 0.004). In multivariable Cox regression analysis, a high GRIm-score was independently associated with an increased cancer-specific mortality risk (hazard ratio [HR] 2.48, 95% confidence interval [CI], 1.31–4.67; *p* = 0.005). Treatment completion was lower in the high GRIm-score group (31.3% vs. 64.4%, *p* = 0.031). **Conclusions**: The GRIm-score serves as an independent prognostic indicator for cancer-specific survival in patients with bladder cancer undergoing curative treatment and is related to therapy completion.

## 1. Introduction

Bladder cancer continues to be a major health challenge globally, ranking among the most prevalent cancers of the urinary system and a top contributor to cancer-related illnesses and deaths worldwide [1,2,3,4,5,6]. Although there have been improvements in surgical methods, radiotherapy, and systemic therapies, the clinical outcomes for patients with bladder cancer treated with the aim of cure remain highly variable [7]. Even among patients with similar pathological stages and treatment plans, survival rates can differ greatly, highlighting the inadequacies of traditional clinicopathological prognostic indicators in effectively assessing individual risk [8,9,10,11,12,13].

Current prognostic markers for bladder cancer, including tumor stage, grade, lymphovascular invasion, and performance status, offer important insights but often fall short of capturing the intricate host–tumor interactions that affect treatment outcomes and long-term survival (8, 11). Consequently, there is an increasing focus on discovering straightforward, reliable, and cost-efficient biomarkers that mirror the systemic biological environment and enhance traditional prognostic models.

Systemic inflammation and nutritional status are crucial factors influencing cancer progression, treatment tolerance, and survival in various solid tumors [14,15,16,17]. Inflammatory processes can facilitate tumor growth, angiogenesis, and immune system evasion, whereas inadequate nutritional status is linked to reduced treatment tolerance and poor cancer outcomes [14,16,17]. Several prognostic indices based on inflammation, which utilize commonly available laboratory parameters such as the neutrophil-to-lymphocyte ratio (NLR), serum albumin, and lactate dehydrogenase (LDH), have shown prognostic significance in a range of cancers, including those of the lung, gastrointestinal tract, and genitourinary system [18,19,20,21].

The Gustave Roussy Immune Score (GRIm-Score) is an inflammation-based composite index that combines serum albumin, LDH, and NLR into a unified prognostic tool [22,23]. The GRIm-Score has been shown to predict survival outcomes across a wide range of solid tumors, including pulmonary adenocarcinoma, colorectal cancer, and small cell lung cancer. [22,24,25,26,27]. Its attractiveness stems from its straightforwardness, reproducibility, and dependence on commonly accessible laboratory tests, making it suitable for regular clinical use [22,23]. Nonetheless, evidence concerning the prognostic significance of the GRIm-score in bladder cancer, particularly in patients receiving curative-intent treatment, remains sparse and inconsistent, reflecting broader difficulties in effectively validating inflammatory and nutritional biomarkers in this disease due to study variability and methodological constraints [28,29].

Crucially, aside from survival rates, the capacity to complete the intended curative treatment plays a vital role in determining the long-term outlook of patients with bladder cancer [30,31]. Treatment interruptions due to toxicity, worsening clinical conditions, or disease progression can undermine the effectiveness of therapy and adversely affect patient outcomes (30). Therefore, biomarkers that can forecast not only survival but also the likelihood of treatment completion could be highly valuable in clinical settings. They would allow for the early detection of high-risk patients who might benefit from enhanced monitoring, supportive care, or tailored treatment plans [32].

The current study aimed to assess the prognostic significance of the GRIm-score on CSS in patients with bladder cancer undergoing treatment with curative intent. Furthermore, we explored the link between the GRIm-score and the completion of treatment, as well as its connection to PFS and OS. By focusing on a clinically uniform group receiving definitive local therapy, this study aims to elucidate the role of the GRIm-Score as a practical prognostic biomarker in the real-world management of bladder cancer.

## 2. Materials and Methods

### 2.1. Study Design and Patient Population

In this retrospective cohort study, patients with bladder cancer confirmed through histopathology were treated with the aim of curing the disease at the Ordu University Training and Research Hospital from October 2010 to April 2025. Participants were eligible if they received bladder-preserving multimodal therapy or underwent radical cystectomy as part of a definitive local treatment plan. This study specifically focused on patients treated with curative-intent methods to create a clinically consistent group with similar therapeutic goals, facilitating a meaningful assessment of CSS.

Treatment completion was defined as receiving the full planned curative protocol without premature discontinuation or receiving ≥80% of the planned treatment intensity, including completion of bladder-preserving multimodal therapy or definitive management with radical cystectomy. Patients who were treated with palliative intent, those with unresectable or metastatic disease, and individuals who did not undergo definitive local therapy were excluded from the analysis.

Individuals with baseline laboratory data available for the calculation of the GRIm-score, which includes serum albumin, LDH, and NLR, were considered eligible for participation. Patients lacking the necessary laboratory values for GRIm-score computation or with incomplete follow-up information were excluded. To minimize confounding from acute inflammatory conditions, patients with clinical suspicion or documented active infection at diagnosis or at the time of baseline laboratory assessment were excluded. Baseline laboratory values used for GRIm-score calculation were obtained within 7 days prior to initiation of definitive treatment and in the absence of clinically apparent infection. Additionally, patients receiving ongoing immunosuppressive therapy—including chronic systemic corticosteroids (≥10 mg/day prednisone equivalent), active immunosuppressive agents (e.g., azathioprine, methotrexate), or immunosuppression related to transplantation or autoimmune disease treatment—were excluded from the study.

Data on clinical, demographic, pathological, and treatment-related factors were retrospectively gathered from electronic medical records. This study adhered to the principles outlined in the Declaration of Helsinki and received approval from the local institutional ethics committee. Owing to the retrospective nature of the study, the requirement for obtaining written informed consent was waived.

### 2.2. Definition of the GRIm-Score

The GRIm score was determined using three commonly measured laboratory parameters: serum albumin, LDH, and NLR. A score was assigned to each of the following conditions: serum albumin less than 3.5 g/dL, LDH greater than 225 U/L, and NLR > 6.0. Patients were divided into two groups based on their total GRIm scores, consistent with previously published research: a low GRIm score group (0–1 points) and a high GRIm score group (2–3 points). Baseline laboratory values used for score calculation were obtained no more than 1 week prior to diagnosis and before initiation of definitive treatment. All laboratory measurements were performed using standardized automated assays in the same institutional laboratory. Hospital-defined reference ranges were applied for all parameters. The LDH cutoff (225 U/L) corresponded to the institutional upper limit of normal.

### 2.3. Clinical Variables and Outcomes

The data collected included age, sex, smoking and alcohol consumption status, existing health conditions, and the Charlson Comorbidity Index (CCI). CCI was determined without considering the bladder cancer diagnosis to prevent score inflation and minimize potential bias associated with including the index disease. Tumor-related variables included pathological stage, histological grade, histopathological subtype, lymphovascular invasion, presence of hydronephrosis, ECOG performance status, and location of the primary tumor. Treatment completion was defined as receiving ≥80% of the planned curative treatment intensity without premature discontinuation.

The main focus was on CSS, defined as the time from diagnosis to death attributable to bladder cancer. Patients who were alive or who died from causes unrelated to bladder cancer were censored at their last follow-up. Follow-up was administratively censored at the study cut-off date of 16 December 2025. Additional outcomes included PFS and treatment completion.

### 2.4. Statistical Analysis

Data analysis was conducted using IBM SPSS Statistics for Windows version 31.0 (IBM Corp., Armonk, NY, USA). Continuous variables were expressed as mean ± standard deviation or median (minimum–maximum), based on their distribution characteristics, while categorical variables were shown as frequencies and percentages. The normality of continuous variables was evaluated by examining the skewness and kurtosis values, with a reference range of ±1.96. To compare the low and high GRIm-score groups, the independent samples *t*-test was used for variables with a normal distribution, and the Mann–Whitney U test was applied for those without. Categorical variables were compared using the chi-square test or Fisher’s exact test, depending on suitability. Survival analysis was performed using the Kaplan–Meier method, with group differences assessed using the log-rank test. Follow-up time was calculated from the date of diagnosis to the date of last clinical contact or death. Patients without an event were administratively censored at the study cutoff date (16 December 2025). Follow-up duration was calculated from the date of diagnosis to the date of last clinical contact or death and summarized using the median and interquartile range. The median survival times and 95% CIs were calculated. Prognostic factors related to cancer-specific survival were analyzed using univariable and multivariable Cox proportional hazard regression models. Variables with a *p*-value < 0.10 in the univariable analyses, along with clinically significant factors, were included in the multivariable model. The treatment period (2010–2017 vs. 2018–2025) was additionally included as a covariate to account for potential era effects.

To prevent multicollinearity, the individual components of the GRIm-Score (albumin, LDH, and NLR) were not included in the multivariable model along with the composite GRIm-Score. Results are presented as hazard ratios (HRs) with 95% confidence intervals. Model discrimination was assessed using Harrell’s concordance index (C-index). Receiver operating characteristic (ROC) curve analysis was used to evaluate the discriminative power of the GRIm-score and its individual components for cancer-specific survival, with the area under the curve (AUC) calculated. In ROC analyses, cancer-specific death was defined as the positive event. For biologically protective variables (e.g., albumin), inverse directionality may yield AUC values below 0.5; therefore, interpretation considered the clinical direction of effect rather than the absolute AUC threshold alone. ROC analyses were conducted for descriptive evaluation of discriminative ability rather than for formal superiority testing or predictive model development. Therefore, AUC values were reported as summary measures of discrimination. Comparative ROC testing (e.g., DeLong test) and calibration analyses were not performed, as these approaches are more appropriate for large-scale predictive model studies and were beyond the scope of the present exploratory prognostic analysis. Correlations between the GRIm-score and its components were assessed using Spearman’s rank correlation coefficient. A two-sided *p*-value < 0.05 was deemed statistically significant. Given the modest sample size—particularly in the high GRIm-score group—propensity score matching was not performed to avoid further loss of statistical power. To assess potential model instability related to the limited sample size and low events-per-variable ratio, internal validation was performed using bootstrap resampling with 1000 iterations for the multivariable Cox proportional hazards model. Bootstrap-adjusted hazard ratios, confidence intervals, and *p*-values were calculated to evaluate the robustness of the estimates.

### 2.5. Use of Generative Artificial Intelligence

ChatGPT (OpenAI GPT-5.1, 2025 release) was used solely for grammatical correction, paraphrasing, and translation support. No generative artificial intelligence tools were used in the study design, data collection, analysis, interpretation, or generation of scientific content. All content was reviewed and approved by the authors.

### 2.6. Ethical Approval

This study was approved by the Ordu University Non-Interventional Clinical Research Ethics Committee (Approval No.: 2025/380; Date: 7 November 2025). All procedures were conducted in accordance with the principles of the Declaration of Helsinki. As this was a retrospective study, the requirement for informed consent was waived by the Ethics Committee.

## 3. Results

### 3.1. Patient Characteristics

**Figure 1:**
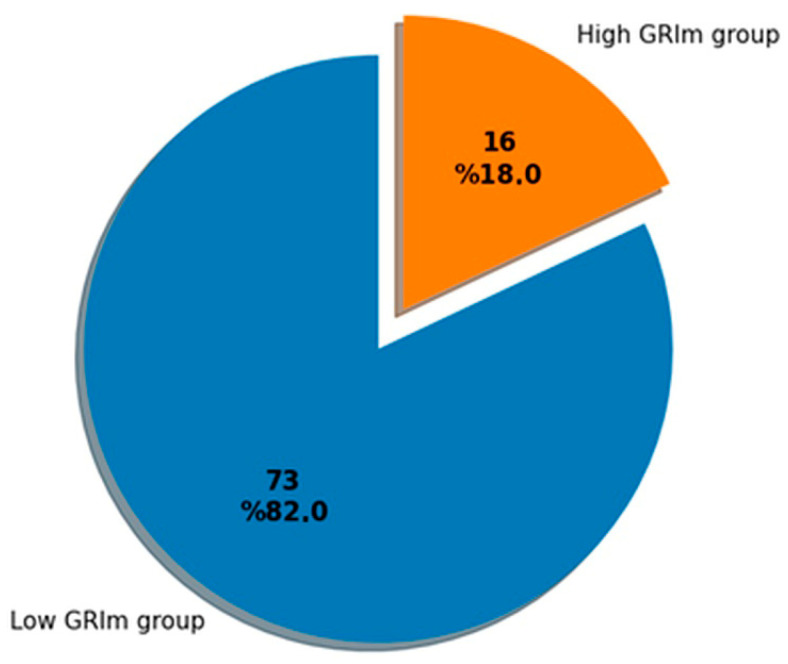
Distribution of GRIm score categories in the study cohort. Most patients belonged to the low GRIm score group (82.0%), whereas a smaller proportion were classified as having a high GRIm score (18.0%).

The analysis included 89 patients with bladder cancer who received treatment to cure the disease. Based on the GRIm-score, 73 patients (82.0%) were placed in the low GRIm-score category (scores 0–1), while 16 patients (18.0%) were categorized in the high GRIm-score group (scores 2–3) (Figure 1).

Table 1 presents a summary of the baseline demographic, clinical, and pathological features categorized by GRIm-score groups. No statistically significant differences were observed between the low- and high-GRIm-score groups in terms of age, sex, smoking habits, alcohol consumption, presence of comorbidities, Charlson Comorbidity Index, or number of medications regularly taken (all *p* > 0.05).

The characteristics related to the tumor, such as the type of treatment (either bladder-preserving therapy or radical cystectomy), T stage, involvement of lymph nodes as seen in radiological assessments, histopathological subtype, histological grade, lymphovascular invasion, presence of hydronephrosis, ECOG performance status, and the location of the primary tumor, were similar between the two groups, with all *p*-values > 0.05.

Conversely, notable variations were found in the laboratory parameters that make up the GRIm-score. Individuals in the high GRIm-score category exhibited considerably lower serum albumin levels and significantly elevated LDH and NLR values compared to those in the low GRIm-score category (all *p* < 0.001).

There was a notable difference in treatment completion rates between the groups, with the low GRIm-score group achieving a higher completion rate than the high GRIm-score group (64.4% vs. 31.3%, *p* = 0.031).

### 3.2. Comparison of Continuous Variables According to GRIm-Score

Table 2 displays the comparisons of continuous variables across the different GRIm-score groups. There were no notable differences between the low and high GRIm-Score groups regarding age, smoking history (measured in pack-years), number of comorbid conditions, Charlson Comorbidity Index, or the number of medications regularly taken (all *p* > 0.05).

In the group with a high GRIm-score, serum albumin levels were notably lower (*p* < 0.001), whereas LDH and NLR values were considerably elevated compared to the group with a low GRIm-score (both *p* < 0.001).

### 3.3. Survival Analyses

**Figure 2:**
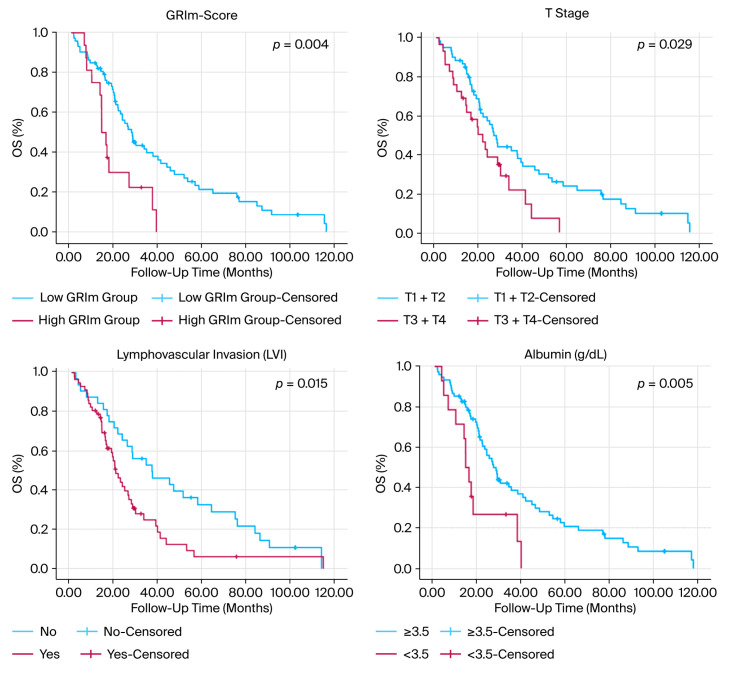

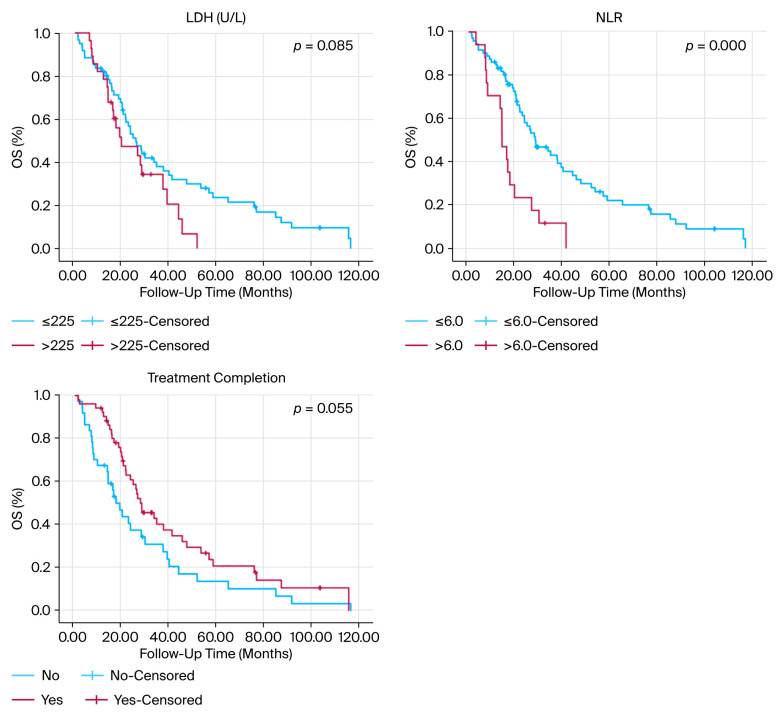
Kaplan–Meier curves for overall survival according to selected clinicopathological and treatment-related variables. Survival differences between groups were assessed using the log-rank test. Curves depict survival stratified by GRIm-score, clinical T stage, lymphovascular invasion status, serum albumin level, LDH level, neutrophil-to-lymphocyte ratio, and treatment completion status. Tick marks indicate censored observations.

The median duration of follow-up was 21.5 months (25th–75th percentile, 13.6–38.3 months). Survival analyses demonstrated significant differences between GRIm-score groups. Patients with a high GRIm-score had markedly shorter PFS (9.42 vs. 16.34 months, *p* = 0.014), OS (15.06 vs. 23.54 months, *p* = 0.033), and CSS (14.07 vs. 27.75 months, *p* = 0.004) compared with those with a low GRIm-score (Figure 2, Table 3). The corresponding five-year CSS rates were 0% and 20%, respectively.

In addition to GRIm-score, higher T stage, presence of lymphovascular invasion, hypoalbuminemia, and elevated NLR were also significantly associated with worse CSS in univariable analyses (all *p* < 0.05; Table 3). Given the modest sample size and heterogeneous follow-up duration, the survival curves showed a substantial reduction in the number of patients at risk at later time points; therefore, estimates in the tail of the Kaplan–Meier curves should be interpreted cautiously.

To evaluate the robustness of the predefined NLR threshold, additional analyses using alternative cutoffs were performed. Sensitivity analyses using alternative NLR thresholds demonstrated consistent prognostic discrimination of the GRIm-score for bothmOS and PFS. (Supplementary Table S1).

### 3.4. Univariable and Multivariable Cox Regression Analyses

Table 4 summarizes the results of the univariable and multivariable Cox regression analyses for cancer-specific survival.

In univariable analyses, a high GRIm-score, advanced T stage (T3–4), lymphovascular invasion, current smoking, hypoalbuminemia, and elevated NLR were significantly associated with worse cancer-specific survival (all *p* < 0.05). Comorbidity burden and treatment completion demonstrated borderline associations (*p* < 0.10).

In the multivariable model, the GRIm-score remained an independent predictor of cancer-specific mortality (HR 2.48, 95% CI 1.31–4.67, *p* = 0.005). Treatment completion was also independently associated with survival (HR 2.47, 95% CI 1.14–5.35, *p* = 0.021), whereas other covariates did not retain statistical significance. Treatment period was not associated with cancer-specific survival (*p* = 0.202).

To minimize multicollinearity, the individual components of the GRIm-score (albumin, LDH, and NLR) were not included simultaneously with the composite score. Model discrimination was moderate, with a Harrell’s C-index of 0.571.

Internal validation using bootstrap resampling (1000 iterations) confirmed the stability of the model estimates. The prognostic effect of the GRIm-score remained statistically significant after bootstrap adjustment (bootstrap *p* = 0.017), supporting the robustness of the association.

**Figure 3:**
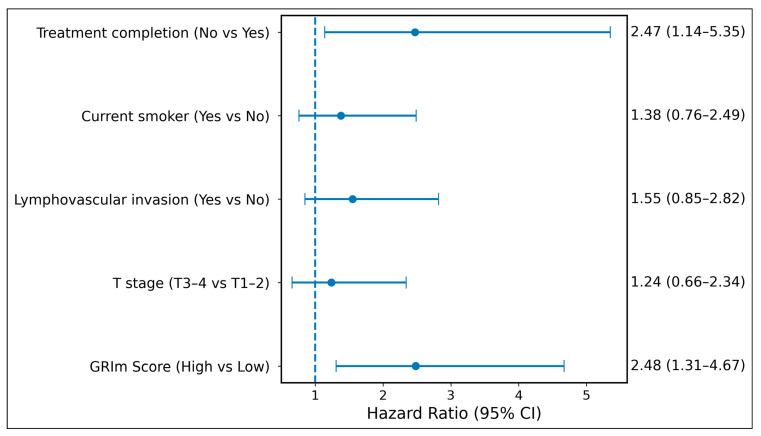
Forest plot showing hazard ratios (HRs) and 95% confidence intervals for factors associated with overall survival. Estimates were obtained from Cox proportional hazards regression analysis. The blue dots represent hazard ratio point estimates, horizontal lines indicate 95% confidence intervals, and the vertical dashed line denotes the reference value (HR = 1.0).

Multivariable hazard ratios and their 95% confidence intervals are illustrated in Figure 3.

### 3.5. ROC and Correlation Analyses

**Figure 4:**
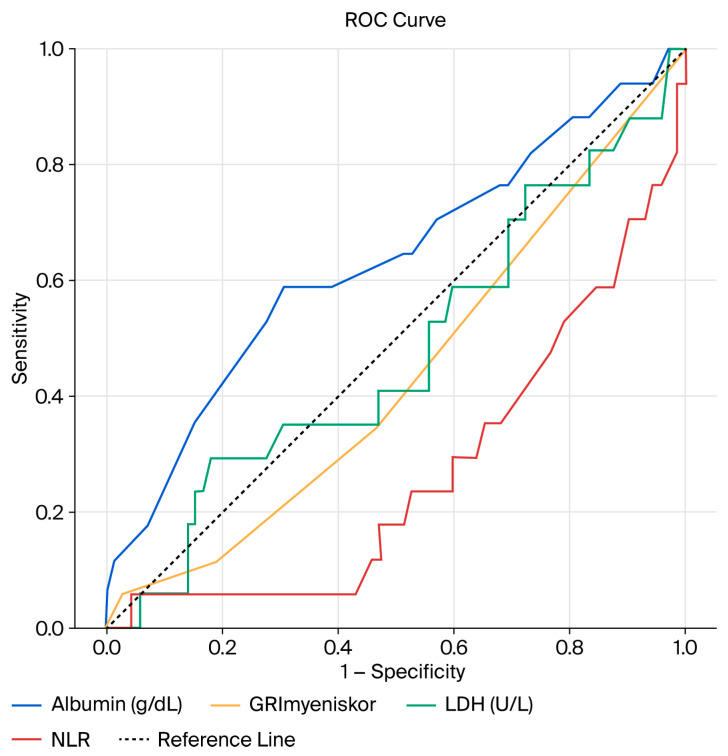
Receiver operating characteristic (ROC) curves of the GRIm-score and its individual components for predicting cancer-specific survival. ROC curves were generated for the GRIm-score, serum albumin level, LDH level, and neutrophil-to-lymphocyte ratio. The diagonal line represents a reference line.

Receiver operating characteristic (ROC) analysis was performed to evaluate the discriminative ability of the GRIm-score and its individual components for cancer-specific survival. Among the evaluated parameters, NLR demonstrated the highest discriminative performance (AUC 0.731, 95% CI 0.598–0.864, *p* = 0.001). In contrast, the GRIm-score, albumin, and LDH showed modest and statistically non-significant AUC values (all *p* > 0.05) (Table 5, Figure 4).

These findings are consistent with the time-dependent nature of oncologic outcomes, as ROC analysis reflects discrimination at a fixed time point, whereas the GRIm-score is primarily intended as a prognostic marker for survival modeling rather than a diagnostic classifier.

**Figure 5:**
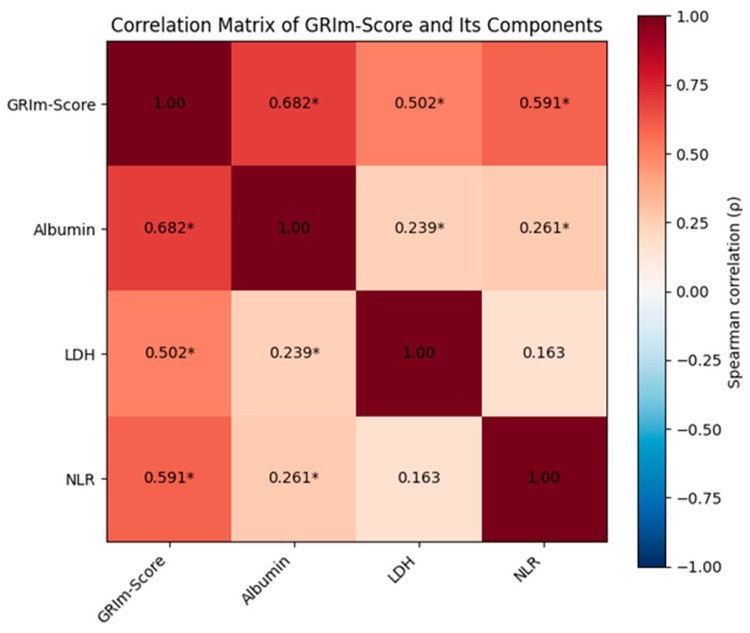
Spearman correlation matrix between the GRIm-score and its components. The cells display Spearman correlation coefficients (ρ) between the GRIm-score, serum albumin, LDH, and neutrophil-to-lymphocyte ratio. Red tones indicate positive correlations, and blue tones indicate negative correlations. Statistically significant correlations are marked with * (*p* < 0.05).

To further assess potential multicollinearity among variables prior to multivariable modeling, correlations between the GRIm-score and its components were examined using Spearman’s rank analysis. Significant positive correlations were observed between the GRIm-score and albumin (ρ = 0.682), LDH (ρ = 0.502), and NLR (ρ = 0.591) (all *p* < 0.001). Weak but statistically significant associations were also detected between albumin and LDH and between albumin and NLR, whereas no significant correlation was found between LDH and NLR (Table 6, Figure 5).

## 4. Discussion

In this real-world cohort of patients with bladder cancer treated with curative intent, the GRIm-score was independently associated with cancer-specific survival. Patients with higher GRIm-scores experienced significantly shorter progression-free, overall, and cancer-specific survival compared with those with lower scores. Importantly, this association persisted after adjustment for established clinicopathological variables, indicating that the GRIm-score may provide additional prognostic information beyond conventional tumor-related factors. Moreover, a higher GRIm-score was associated with a lower likelihood of treatment completion, suggesting that the score may reflect not only prognosis but also clinical vulnerability and treatment tolerability.

Beyond confirming the prognostic value of inflammation-based biomarkers, our study offers several clinically relevant contributions. Unlike many previous reports conducted in metastatic or immunotherapy-treated settings, our analysis focused exclusively on patients treated with curative intent, a population in which accurate pre-treatment risk stratification is particularly important for long-term disease control. In this context, the observed relationship between the GRIm-score and both survival outcomes and treatment completion highlights the potential practical utility of this score in routine clinical decision-making.

The GRIm-score was originally developed for patient stratification in early-phase clinical trials and incorporates LDH, serum albumin, and NLR—markers reflecting systemic inflammation, metabolic tumor burden, and nutritional status [33,34]. Each of these components has independently demonstrated prognostic relevance across malignancies. In particular, elevated NLR has consistently been associated with adverse outcomes in multiple cancers [35,36] and meta-analyses in bladder cancer have linked high pretreatment NLR to poorer survival after radical cystectomy [37,38]. Similarly, increased LDH levels have been correlated with unfavorable outcomes in urothelial carcinoma [39,40] while hypoalbuminemia has been associated with malnutrition, frailty, postoperative complications, and reduced survival [41,42]. Taken together, these findings provide a strong biological rationale for integrating inflammatory and nutritional parameters into a composite index rather than relying solely on tumor-centered prognostic factors.

Several other inflammation-based indices, including the Glasgow Prognostic Score (GPS), modified GPS, Prognostic Nutritional Index (PNI), and Systemic Immune–Inflammation Index (SII), have demonstrated prognostic value in urothelial and other solid tumors [18,36,43]. However, most rely on a limited number of parameters that primarily reflect either inflammation or nutritional status alone. In contrast, the GRIm-score simultaneously incorporates complementary biological domains—systemic inflammation, tumor-related metabolic activity, and nutritional reserve—thereby providing a broader representation of host–tumor interaction [26,44]. This multidimensional structure may partly explain the independent prognostic performance observed in our cohort and supports its potential complementary use alongside established clinicopathological factors in routine practice.

An apparent discrepancy was observed between the moderate discrimination seen in ROC analyses and the significant associations detected in time-to-event models. This difference likely reflects methodological rather than biological inconsistency. ROC analysis evaluates outcomes at fixed time points, whereas survival models account for the time-dependent nature of oncologic events. Because the GRIm-score reflects dynamic systemic processes such as inflammation, immune dysregulation, and nutritional decline, it may be more appropriately interpreted as a prognostic marker for longitudinal risk rather than a diagnostic classifier at a single time point [16,45,46].

In addition to survival prediction, the association between a high GRIm-score and reduced treatment completion carries direct clinical implications. Completion of planned multimodal or radical therapy is a key determinant of cure in localized bladder cancer, particularly in bladder-preserving trimodal strategies [47,48,49,50]. Patients with elevated scores may have diminished physiological reserve, heightened systemic inflammation, and greater susceptibility to treatment-related toxicity, which may predispose them to interruptions or early discontinuation of therapy [37,39,51,52]. From a practical perspective, the GRIm-score may therefore help identify patients who could benefit from early nutritional optimization, closer toxicity monitoring, or individualized supportive care [53,54].

Our findings are consistent with emerging evidence demonstrating the prognostic relevance of the GRIm-score in other urothelial cancer settings, including patients treated with immune checkpoint inhibitors, where higher scores have been associated with poorer outcomes [55]. The consistency of these observations across disease contexts reinforces the broader concept that host inflammatory and nutritional status plays a critical role in determining oncologic outcomes [15,56]. Prospective multicenter studies are warranted to externally validate these results and to determine whether incorporation of the GRIm-score into multivariable risk models or nomograms [25] could further improve patient stratification.

Although subgroup or interaction analyses according to treatment modality or disease stage might provide additional insights, the modest sample size—particularly within the high-risk group—limited the feasibility of reliable stratified analyses. Larger cohorts will be required to explore potential effect modification across clinical subgroups.

From a clinical standpoint, the GRIm-score represents a simple, inexpensive, and readily accessible tool derived from routine laboratory parameters. Rather than replacing established prognostic systems, it may provide complementary information that assists clinicians in identifying vulnerable patients and tailoring supportive or therapeutic strategies accordingly.

## 5. Limitations

This study has several limitations that should be considered when interpreting the findings. First, its retrospective single-center design may limit generalizability and introduce potential selection bias. Second, although we focused on a relatively homogeneous curative-intent population, the overall sample size—particularly the number of patients in the high GRIm-score group—was modest, which may have reduced statistical power and increased the risk of model instability. To mitigate this concern, internal validation using bootstrap resampling was performed, and the results remained consistent; however, external validation in larger prospective multicenter cohorts is required.

Third, the components of the GRIm-score were assessed only at baseline. Temporal changes in inflammatory and nutritional parameters during treatment were not evaluated and may provide additional prognostic information. As with all retrospective observational studies, residual confounding from unrecognized inflammatory conditions or comorbidities cannot be completely excluded, and causal inferences cannot be established.

Additionally, data on certain potential confounders—including prior intravesical therapies, detailed body composition measures such as BMI, and comprehensive toxicity grading—were not consistently available. Serum albumin, although routinely used as a nutritional marker, is nonspecific and may also be influenced by hepatic, renal, or systemic comorbidities independent of cancer-related inflammation. Furthermore, evolving treatment strategies over the long study period may have introduced clinical heterogeneity despite adjustment for treatment era.

Despite these limitations, the consistency of the observed associations across multiple survival endpoints and the use of robust statistical approaches support the clinical relevance of our findings.

## 6. Conclusions

In this real-world cohort of patients with bladder cancer treated with curative intent, the GRIm-score demonstrated independent prognostic value for cancer-specific survival. Patients with higher scores experienced poorer survival outcomes and were less likely to complete planned treatment, suggesting increased clinical vulnerability. By integrating routinely available markers of systemic inflammation and nutritional status, the GRIm-score represents a simple and accessible tool that may complement traditional clinicopathological risk assessment. Prospective, multicenter validation is warranted before routine clinical implementation.

## Figures and Tables

**Table 1:** Comparison of demographic, clinical, and pathological characteristics according to GRIm-score groups.

Variables	Category	Overall n (%) (n: 89)	Low GRIm-Score n (%), (n: 73)	High GRIm-Score n (%), (n: 16)	*p* Value
Age	≤80 years	44 (49.4)	37 (50.7)	7 (43.8)	0.821
	>80 years	45 (50.6)	36 (49.3)	9 (56.3)	
Sex	Female	17 (19.1)	12 (16.4)	5 (31.3)	0.311
	Male	72 (80.9)	61 (83.6)	11 (68.8)	
Smoking status	Never	24 (27.0)	19 (26.0)	5 (31.3)	0.875
	Former	51 (57.3)	42 (57.5)	9 (56.3)	
	Current	14 (15.7)	12 (16.4)	2 (12.5)	
Alcohol consumption	Never	65 (73.0)	52 (71.2)	13 (81.3)	0.612
	Former	24 (27.0)	21 (28.8)	3 (18.8)	
Comorbidity	Absent	15 (16.9)	12 (16.4)	3 (18.8)	1.000
	Present	74 (83.1)	61 (83.6)	13 (81.3)	
HT	Absent	31 (34.8)	25 (34.2)	6 (37.5)	1.000
	Present	58 (65.2)	48 (65.8)	10 (62.5)	
DM	Absent	70 (78.7)	57 (78.1)	13 (81.3)	1.000
	Present	19 (21.3)	16 (21.9)	3 (18.8)	
COPD	Absent	70 (78.7)	58 (79.5)	12 (75.0)	0.955
	Present	19 (21.3)	15 (20.5)	4 (25.0)	
ASCVD/CHF	Absent	52 (58.4)	43 (58.9)	9 (56.3)	1.000
	Present	37 (41.6)	30 (41.1)	7 (43.8)	
Curative treatment	Bladder-preserving	70 (78.7)	56 (76.7)	14 (87.5)	0.537
	Radical cystectomy	19 (21.3)	17 (23.3)	2 (12.5)	
T stage	T1–T2	60 (67.4)	50 (68.5)	10 (62.5)	0.866
	T3–T4	29 (32.6)	23 (31.5)	6 (37.5)	
Radiological LN involvement	LN negative	64 (71.9)	54 (74.0)	10 (62.5)	0.537
	LN positive	25 (28.1)	19 (26.0)	6 (37.5)	
Histopathology	Pure urothelial	66 (74.2)	55 (75.3)	11 (68.8)	0.818
	Variant histology	23 (25.8)	18 (24.7)	5 (31.3)	
Histological grade	Low (0–II)	11 (12.4)	11 (15.1)	0 (0.0)	0.215
	High (III–IV)	78 (87.6)	62 (84.9)	16 (100.0)	
Lymphovascular invasion	Absent	32 (36.0)	28 (38.4)	4 (25.0)	0.471
	Present	57 (64.0)	45 (61.6)	12 (75.0)	
Hydronephrosis	Absent	67 (75.3)	57 (78.1)	10 (62.5)	0.323
	Present	22 (24.7)	16 (21.9)	6 (37.5)	
ECOG performance status	0–1	47 (52.8)	42 (57.5)	5 (31.3)	0.103
	2–4	42 (47.2)	31 (42.5)	11 (68.8)	
Primary tumor location	Lateral wall	66 (74.2)	56 (76.7)	10 (62.5)	0.401
	Anterior–Posterior	13 (14.6)	9 (12.3)	4 (25.0)	
	Dome–Trigone	10 (11.2)	8 (11.0)	2 (12.5)	
Albumin (g/dL)	≥3.5	75 (84.3)	70 (95.9)	5 (31.3)	<0.001 **
	<3.5	14 (15.7)	3 (4.1)	11 (68.8)	
LDH (U/L)	≤225	61 (68.5)	58 (79.5)	3 (18.8)	<0.001 **
	>225	28 (31.5)	15 (20.5)	13 (81.3)	
NLR	≤6.0	72 (80.9)	67 (91.8)	5 (31.3)	<0.001 **
	>6.0	17 (19.1)	6 (8.2)	11 (68.8)	
Neoadjuvant chemotherapy	Not received	80 (89.9)	66 (90.4)	14 (87.5)	1.000
	Received	9 (10.1)	7 (9.6)	2 (12.5)	
Treatment completion	Not completed	37 (41.6)	26 (35.6)	11 (68.8)	0.031 *
	Completed	52 (58.4)	47 (64.4)	5 (31.3)	
Recurrence	Absent	31 (34.8)	27 (37.0)	4 (25.0)	0.534
	Present	58 (65.2)	46 (63.0)	12 (75.0)	
Mortality	No	17 (19.1)	15 (20.5)	2 (12.5)	0.696
	Yes	72 (80.9)	58 (79.5)	14 (87.5)	

Data are presented as n (%). Group comparisons were performed using the chi-square test or Fisher’s exact test, as appropriate. *p* < 0.05 was considered statistically significant. Low GRIm-score = 0–1; High GRIm-score = 2–3. COPD, chronic obstructive pulmonary disease; ASCVD, atherosclerotic cardiovascular disease; CHF, congestive heart failure; DM, diabetes mellitus; HT, hypertension; ECOG, Eastern Cooperative Oncology Group; GRIm-score, Gustave Roussy Immune Score; LDH, lactate dehydrogenase; NLR, neutrophil-to-lymphocyte ratio; LN, lymph node. *p* < 0.05 was considered statistically significant (*p* < 0.05 *, *p* < 0.01 **).

**Table 2:** Comparison of descriptive statistics of continuous variables according to GRIm-score groups.

Variables	Overall Mean ± SD/Median (Min–Max)	Low GRIm-Score Mean ± SD/Median (Min–Max)	High GRIm-Score Mean ± SD/Median (Min–Max)	*p* Value
Age (years) ^t^	79.04 ± 8.26 80.05 (60.04–97.18)	78.95 ± 8.45 79.95 (60.04–93.56)	79.46 ± 7.58 80.22 (66.93–97.18)	0.823
Smoking exposure (pack-years) ^t^	34.00 ± 28.29 35.00 (0.00–120.00)	35.26 ± 29.17 35.00 (0.00–120.00)	28.25 ± 23.87 30.00 (0.00–67.00)	0.372
Number of comorbidities ^t^	1.94 ± 1.26 2.00 (0.00–5.00)	1.92 ± 1.22 2.00 (0.00–5.00)	2.06 ± 1.44 3.00 (0.00–4.00)	0.679
Charlson Comorbidity Index (CCI) ^t^	4.80 ± 1.69 5.00 (1.00–9.00)	4.67 ± 1.65 5.00 (1.00–8.00)	5.38 ± 1.82 5.50 (3.00–9.00)	0.133
Number of regularly used medications ^t^	3.21 ± 2.23 3.00 (0.00–11.00)	3.14 ± 2.23 3.00 (0.00–11.00)	3.56 ± 2.31 4.50 (0.00–7.00)	0.493
Albumin (g/dL) ^t^	3.96 ± 0.47 4.00 (2.80–4.80)	4.09 ± 0.39 4.02 (3.00–4.80)	3.39 ± 0.40 3.40 (2.80–4.30)	<0.001 **
LDH (U/L) ^z^	197.38 ± 73.53 184.00 (80.00–495.00)	182.85 ± 64.90/177.00 (80.00–436.00)	263.69 ± 76.08 252.00 (167.00–495.00)	<0.001 **
NLR ^z^	5.63 ± 12.73 3.50 (1.20–120.00)	3.61 ± 2.97 3.00 (1.20–25.00)	14.87 ± 28.25 8.05 (2.10–120.00)	<0.001 **
Progression-free survival (PFS, months) ^z^	21.54 ± 21.28 14.96 (0.56–103.59)	23.93 ± 22.71 16.34 (0.56–103.59)	10.62 ± 5.04 9.42 (4.04–20.61)	0.014 *
Overall survival (OS, months) ^z^	30.64 ± 26.85 21.47 (1.28–116.75)	33.43 ± 28.55 23.54 (1.28–116.75)	17.93 ± 10.39 15.06 (6.15–39.06)	0.033 *

Data are presented as mean ± standard deviation or median (minimum–maximum), as appropriate. ^t^ Normally distributed variables were compared using the independent samples *t*-test. ^z^ Non-normally distributed variables were compared using the Mann–Whitney *U* test. Low GRIm-score was defined as 0–1 points, and high GRIm-score as 2–3 points. *p* < 0.05 was considered statistically significant (*p* < 0.05 *, *p* < 0.01 **).

**Table 3:** Median survival times and 5-year cancer-specific survival (CSS) rates according to clinical and pathological variables.

Variables	Category	Median (Months)	Std. Error	95% CI (Lower–Upper)	*p* Value	5-Year CSS (%)
Age	≤80 years	24.66	3.88	17.05–32.27	0.674	22
	>80 years	26.20	3.43	19.48–32.93		15
GRIm-Score	Low GRIm	27.75	2.98	21.92–33.58	0.004 **	20
	High GRIm	14.07	2.01	10.14–18.00		0
Sex	Female	28.31	12.06	4.68–51.94	0.809	22
	Male	24.66	2.72	19.33–29.98		15
Smoking status	Never	34.62	6.24	22.39–46.85	0.081	25
	Former	23.51	3.17	17.29–29.73		15
	Current	19.33	7.90	3.84–34.82		0
Alcohol consumption	Never	21.63	3.69	14.41–28.86	0.449	18
	Former	28.08	2.63	22.92–33.23		16
Comorbidity	Absent	41.10	16.82	8.13–74.07	0.052	29
	Present	23.54	3.18	17.30–29.78		15
Curative treatment	Bladder-preserving	21.47	2.34	16.88–26.06	0.424	20
	Radical cystectomy	29.69	3.22	23.38–35.99		15
T stage	T1–T2	26.56	2.15	22.34–30.79	0.029	22
	T3–T4	21.47	4.07	13.50–29.44		0
Radiological LN status	Negative	28.08	4.48	19.29–36.86	0.106	19
	Positive	19.00	2.21	14.67–23.33		14
Histopathology	Pure urothelial	23.54	3.73	16.23–30.85	0.500	19
	Variant	26.20	4.33	17.71–34.69		16
Histological grade	Low (0–II)	53.36	24.73	4.88–101.83	0.464	19
	High (III–IV)	23.54	3.15	17.36–29.72		13
Lymphovascular invasion	Absent	37.32	11.52	14.74–59.89	0.015 *	30
	Present	20.48	2.15	16.27–24.69		10
Hydronephrosis	Absent	26.20	2.61	21.08–31.32	0.414	21
	Present	21.63	4.62	12.57–30.69		9
ECOG performance status	0–1	25.84	3.08	19.80–31.88	0.154	19
	2–4	23.54	5.01	13.73–33.35		11
Primary tumor location	Lateral wall	25.84	4.08	17.84–33.84	0.228	17
	Ant.–Post.	16.50	3.96	8.74–24.26		14
	Dome–Trigone	37.32	8.70	20.26–54.37		19
Albumin (g/dL)	≥3.5	26.56	2.38	21.91–31.22	0.005	19
	<3.5	14.04	1.42	11.27–16.81		0
LDH (U/L)	≤225	25.84	2.91	20.15–31.54	0.085	21
	>225	19.73	7.00	6.00–33.45		0
NLR	≤6.0	28.31	4.76	18.97–37.64	<0.001	19
	>6.0	14.07	1.40	11.33–16.81		0
Neoadjuvant chemotherapy	Not received	24.66	3.32	18.14–31.17	0.667	15
	Received	27.75	12.89	2.48–53.02		40
Treatment completion	Not completed	19.00	2.72	13.67–24.33	0.055	12
	Completed	28.31	4.10	20.27–36.35		22

Median survival times were estimated using the Kaplan–Meier method. *p* values were derived from the log-rank test. *p* < 0.05 was considered statistically significant (*p* < 0.05 *, *p* < 0.01 **). CSS, cancer-specific survival.

**Table 4:** Results of univariable and multivariable Cox regression analyses for cancer-specific survival.

Variables	Univariable Analysis			Multivariable Analysis		
	*p*	HR (Exp(B))	95% CI	*p*	HR (Exp(B))	95% CI
GRIm-Score (High vs. Low)	0.005	2.42	1.31–4.49	0.005	2.48	1.31–4.67
T stage (T3–4 vs. T1–2)	0.031	1.78	1.05–3.01	0.508	1.24	0.66–2.34
Lymphovascular invasion (Yes vs. No)	0.017	1.85	1.12–3.07	0.152	1.55	0.85–2.82
Comorbidity (Yes vs. No)	0.056	1.92	0.98–3.73	–	–	–
Smoking status (overall)	0.087	–	–	–	–	–
Former smoker	0.176	1.46	0.84–2.53	0.069	–	–
Current smoker	0.028	2.29	1.09–4.79	0.292	1.38	0.76–2.49
Treatment completion (No vs. Yes)	0.057	0.63	0.40–1.01	0.021	2.47	1.14–5.35
Albumin (<3.5 vs. ≥3.5 g/dL)	0.007	2.44	1.28–4.67	–	–	–
LDH (>225 vs. ≤225 U/L)	0.087	1.59	0.93–2.70	–	–	–
NLR (>6.0 vs. ≤6.0)	<0.001	2.87	1.59–5.18	–	–	–

Variables with *p* < 0.10 in the univariable analysis and those considered clinically relevant were included in the multivariable Cox regression model. To avoid multicollinearity, the individual components of the GRIm-score (albumin, LDH, and NLR) were not simultaneously included in the multivariable model together with the composite GRIm-score. HR, hazard ratio; CI, confidence interval; GRIm-score, Gustave Roussy Immune Score; LDH, lactate dehydrogenase; NLR, neutrophil-to-lymphocyte ratio. The discriminative ability of the multivariable model was moderate (Harrell’s C-index = 0.571). Variables included in the multivariable model: GRIm-score, T stage, lymphovascular invasion, comorbidity, smoking status, treatment completion, albumin, LDH and NLR. To avoid model overfitting given the limited sample size, the number of covariates included in the multivariable model was restricted to clinically relevant variables.

**Table 5:** ROC analysis results of GRIm-score components for predicting cancer-specific survival.

Test Variable	AUC	Std. Error	*p* Value	%95 CI (Lower–Upper)
GRIm-Score	0.562	0.077	0.418	0.412–0.712
Albumin (g/dL)	0.368	0.081	0.102	0.209–0.526
LDH (U/L)	0.523	0.081	0.777	0.365–0.681
NLR	0.731	0.068	0.001	0.598–0.864

Receiver operating characteristic (ROC) analysis was performed to evaluate the discriminative ability of the GRIm-score and its individual components for cancer-specific survival. AUC, area under the curve; CI, confidence interval; LDH, lactate dehydrogenase; NLR, neutrophil-to-lymphocyte ratio. A *p* value < 0.05 was considered statistically significant.

**Table 6:** Results of correlation analysis between the GRIm-score and its components.

Variables	Statistic	GRIm-Score	Albumin (g/dL)	LDH (U/L)	NLR
GRIm-Score	r	1	0.682	0.502	0.591
	*p*	.	<0.001 **	<0.001 **	<0.001 **
Albumin (g/dL)	r	0.682	1	0.239	0.261
	*p*	<0.001 **	.	0.024 *	0.013 *
LDH (U/L)	r	0.502	0.239	1	0.163
	*p*	<0.001 **	0.024 *	.	0.126
NLR	r	0.591	0.261	0.163	1
	*p*	<0.001 **	0.013 *	0.126	.

Correlations between variables were assessed using Spearman’s rank correlation coefficient (ρ). *p* < 0.05 (*) and *p* < 0.001 (**) were considered statistically significant. GRIm-score, Gustave Roussy Immune Score; LDH, lactate dehydrogenase; NLR, neutrophil-to-lymphocyte ratio.

## Data Availability

The datasets generated and/or analyzed during the current study are not publicly available because of patient privacy and institutional data protection policies but are available from the corresponding author upon reasonable request.

## Supplementary Materials

The following supporting information can be downloaded at: https://www.mdpi.com/article/10.3390/diagnostics16040574/s1, Table S1: Median Overall Survival (OS) and Progression-Free Survival (PFS) According to GRIm Score Using Alternative NLR Cut-offs.

## Abbreviations

AUC	Area Under the Curve
BC	Bladder Cancer
CCI	Charlson Comorbidity Index
CI	Confidence Interval
CSS	Cancer-Specific Survival
ECOG	Eastern Cooperative Oncology Group
EGFR	Epidermal Growth Factor Receptor
GRIm-score (GRImS)	Gustave Roussy Immune Score
HR	Hazard Ratio
LDH	Lactate Dehydrogenase
NLR	Neutrophil-to-Lymphocyte Ratio
OS	Overall Survival
PFS	Progression-Free Survival
ROC	Receiver Operating Characteristic
TURBT	Transurethral Resection of Bladder Tumor
WHO	World Health Organization
