# Supplementary material for: Prognostic Impact of the Gustave Roussy Immune Score on Cancer-Specific Survival and Treatment Completion in Patients with Bladder Cancer
Source: Diagnostics (Basel). 2026 Feb 14;16(4):574. doi: 10.3390/diagnostics16040574 (PMC12939023; doi:10.3390/diagnostics16040574)
Supplement: Supplementary file 1 [file diagnostics-16-00574-s001.zip › diagnostics-4115595-supplementary.pdf]

Supplementary Table S1. Median Overall Survival (OS) and Progression-Free Survival (PFS) According to GRIm Score Using Alternative NLR Cut-offs

NLR Cut-off	Outcome	GRIm Risk Group	Median Survival (months)	95% Confidence Interval	Log-rank p
**NLR ≥5**	OS	Low (0–1)	28.1	22.8–33.4	<0.001
		High (2–3)	14.1	9.7–18.5	
**NLR ≥5**	PFS	Low (0–1)	20.4	12.7–28.2	0.002
		High (2–3)	9.6	3.9–15.2	
**NLR ≥7**	OS	Low (0–1)	27.7	22.5–33.0	0.010
		High (2–3)	16.0	12.9–19.2	
**NLR ≥7**	PFS	Low (0–1)	19.3	11.1–27.4	0.020
		High (2–3)	11.5	7.6–15.4	
